# Shackles and Chains: Some Concluding Thoughts

**DOI:** 10.1007/978-3-030-54871-1_9

**Published:** 2020-10-31

**Authors:** Claire Hilton

**Affiliations:** Centre for the History of Emotions, Queen Mary University of London, London, UK

## Abstract

Many of the themes running through this book have parallels today, associated with stigma, austerity, deaths from preventable causes, and under-provision of services from which mentally unwell people could benefit. Metaphorical shackles and chains link then and now. At the beginning of the war, speculation and hope that victory would be within easy reach informed asylum planning. Decisions justifiable on that basis, but without compensatory adjustments later in the war, created harsher asylum regimes. Public support and special provision for mentally disturbed soldiers was heartening, but it highlighted the undignified standards of care provided for mentally disturbed civilians. Despite the problems in the asylums, there were also many dedicated and kind staff. Kindness was assumed so it was not noteworthy: it usually only came to light incidental to some other matter.

## Then and Now

The past has continuity with the present and the future. The present can assist in formulating questions to help investigate the past, and the past can shed light on current policy, practice and culture, and inform debate on future health services.^1^ Iron shackles and chains, once used to restrain mentally disturbed patients in asylums in England, were replaced by leather and strong cloth many years before the First World War. Today’s shackles and chains are metaphorical, like heavy-duty polymer threads, nearly invisible but resistant to breakage. They limit the lives of people with severe enduring mental illness who live in the community. They also tie government, public and professionals to concepts and values from the past, such as the acceptability of resourcing mental health and social care services which barely reach the levels needed, and rarely exceed them. Threads also link research challenges past and present: neuroscience has still not disclosed answers to allow us to prevent or cure schizophrenia, bipolar (manic-depressive) and other disabling psychiatric disorders, despite an ever-increasing grasp of their underlying causative mechanisms. These age-old challenges continue to spur on researchers, to overcome obstacles and to achieve scientific, pharmacological and clinically significant breakthroughs. Psychiatrists and others supporting patients over the last century have worked amid ongoing clinical and scientific uncertainty. They have aimed to identify the best pathways to alleviate their patients’ suffering while grappling with shifting concepts, hypotheses and disease classifications, in the context of practice shaped by national and local events and government policy endeavours. Historians and clinicians need to be wary of disparaging our forebears’ practices and understanding of scientific evidence through our lens of hindsight, just as we hope that future generations will analyse dispassionately the strengths and deficits of our less than perfect knowledge and its clinical application.

Other continuities bind past and present. Asylums had walls of stone, bricks and mortar and patients lived communally in barrack-like buildings segregated by gender. The system of community care since the asylums closed lacks physical walls, but metaphorical ones exist. People with severe chronic mental illness today have more privacy, personal autonomy and independence than those a century ago, and many respond well to new medical, psychological or social treatment approaches. But many are unemployed, have poor physical health, receive inadequate social welfare payments and insufficient support from suitably trained staff, and are separated from their families and from broader community involvement. Asylum care had, and community care has, downsides and upsides. Both need to be understood in the distinct cultural frameworks of their times and in the broader context of societal values, including about institutions, illness, treatment, care, autonomy, independence, risk and protection.

The Board of Control (“the Board”) and some individual psychiatrists, notably Charles Mercier, William Stoddart and Lionel Weatherly,^2^ advocated gold standards of humane treatment leading at best to recovery, otherwise to a fulfilling life for those with the most severe chronic mental disorders who were unable, according to clinical reasoning and lunacy law at the time, to leave the asylums. Best practice was recognised, but emulated insufficiently, and asylums spanned a range of standards from admirable to appalling, as community care does today. Despite shared ideals between the asylums and community care, particularly the importance of people with chronic psychiatric disorders having as near normal lives as possible, some constructive asylum practices have been lost in the community care system. To take the example of employment: paid or unpaid meaningful occupation has long been considered helpful in the context of mental disorders to build confidence and self-esteem and improve health and wellbeing. In praiseworthy asylums up to 90 per cent of patients were engaged in some sort of daily work in 1914^3^ which could be linked to the skills they acquired pre-admission.^4^ By comparison, in 2013, when the UK working-age employment rate was 71 per cent,^5^ only 10–15 per cent of people with schizophrenia were in employment although many more could, and wanted to, work.^6^ This is a modern tragedy.

By the First World War, model asylum practices embracing humane and individually focussed psycho-social treatment had waned and care had become increasingly custodial, but even then, patients recovered and were discharged. Asylums were too often overcrowded, understaffed, unhygienic and warehouse like. This social warehousing was a consequence of long-term legal and financial constraints linked to values, knowledge and attitudes of professionals, policy leaders and the general public. Once it became accepted as normal, it perpetuated as a convenient way to proceed, unquestioned by most people. Similarly, when standards worsened, associated with wartime austerity, too often the state of affairs was accepted as the new normal and created little protest. When Lionel Shadwell, for example, inspected Claybury and noted high death rates, he was not alarmed as they were from “natural and ordinary” causes of the sort prevalent pre-war.^7^ The continuation of pre-existing trends could be ignored, in contrast to the response when something unexpected appeared, whether shell-shock, or the Covid-19 pandemic as I write. Something new demands attention, but concurrently can expose the realities faced by vulnerable people living in deprived circumstances, whether pauper lunatics in the asylums of the past, or people living in poverty, or under community care, or in institutions today. At a time of crisis, long-lasting deficits temporarily become newsworthy.^8^ The risk is that, after the crisis, in a period of reconstruction, the deficits fall back to their pre-crisis low priority. This happened to the asylums, perpetuating injustices and inequalities. We are yet to see what will happen after the Covid-19 pandemic.

The wartime asylums, and limitations of community care today, demonstrate provision of health and social care services which fail to meet the needs of many of those whom they are meant to serve. During the war, the asylum leadership waited as long as they dared, arguably too long, before asking for more resources to prevent deterioration in disastrously poor asylum standards. Today, despite admirable campaigning by patient-led groups, voluntary organisations, the Royal College of Psychiatrists and others, in the present climate of austerity the needs of some of the most seriously mentally ill people are side-lined.^9^ Dangers exist when complacency prevails within a mental health service system, then and now. As Adrian James wrote in his forward to this book: “Continued self-reflection and challenge are vital. We could still do so much more.”

## Leadership: Attitudes and Standards

At the beginning of the war, speculation and hope that victory would be within easy reach informed asylum planning. Decisions made on that basis for a short-term national emergency may have been justifiable, but as time went on without compensatory adjustments for the prolonged duration, the asylum environment became harsher. Food declined in quality and quantity; care was more custodial and less rehabilitative; fewer and less well-trained staff were employed, often on temporary contracts; and many patients were moved from their “home” asylum to other overcrowded asylums at a distance, to make way for military casualties. The Board claimed at the beginning of the war that compromises in the asylums would not be detrimental to patients’ well-being. This did not hold.

Despite the Board “policing” the asylums, it only had authority to advise and persuade medical superintendents and “visiting” committees (VCs) to make improvements. Responses of VCs fluctuated, arguably associated with insufficient knowledge about health and illness and the full purpose and intricacies of asylum function. Their level of activity appeared to be that which was the minimum required to conform to the Lunacy Act or other mandatory or closely monitored directives. They frequently attributed inactivity to financial constraints, which became more burdensome associated with wartime price rises. The asylum system was torn between how it assisted with wartime objectives and how it provided for the patients’ needs. For the leadership, the war took priority. It was simpler to demonstrate patriotism and to go with public and government sentiment, rather than advocate for patients who were not valued by society.

At all levels, staff defended and justified their decision making, or passed the buck up or down the ladder, deflecting responsibilities away from themselves. The Board passed its dilemmas to the Home Office, War Office, Ministry of Food and other Whitehall bodies. The VCs passed theirs to the Board or medical superintendent, or to lower ranks of staff, who passed their discontent onto the patients. Risk of dismissal deterred low ranks of staff from criticising the asylum,^10^ and some took out their frustration on patients by “ rough handling” them, and then justifying their actions as being reasonable responses to the patients’ needs. Theories about patients being inevitably unreliable due to their insanity, and being susceptible to physical injury, such as by having fragile bones, helped staff avoid punishment for their heavy handedness. Complaints made by patients concerning their care, or by their relatives on their behalf, were typically ignored, interpreted as signs of mental derangement. Patients who reported maltreatment were liable to retribution. For them, it came in the form of further physical or psychological abuse from the staff on the wards. If VCs paid attention to the complaints, investigations were likely to be undertaken behind closed doors within the institution by senior people with potential conflicts of interest.^11^ Rough handling was not unique to the wartime asylums: abuse by staff in hospitals and other residential institutions caring for vulnerable people has continued through the twentieth century and into the twenty-first.^12^

According to Adolph Meyer, the “rigidly moralising attitude” of “Anglo-Saxon” communities aimed to regulate and remove, rather than understand, mental disorders.^13^ Removing mentally disturbed people to asylums placed them out of sight and out of mind, minimising community conscience and public interest and any sense of responsibility towards them as fellow human beings. Removing them also assisted with concealing institutional inadequacies and revealing as little as possible to the public. Little external oversight, interest and communication enshrined the asylum system, protected the reputations of institutions and leadership, and added to public perceptions of stigma and fear of asylums, of insanity and of those suffering from it. Theories of degeneration or hereditary predisposition to insanity added to overall negativity (but did not necessarily deter doctors from treating patients so labelled). It is unsurprising, amid the secrecy, fear and negativity, that the Boards of Guardians, who took decisions on behalf of their local communities, were reluctant to pay more to the asylums for the patients’ care.

In contrast to a rigid asylum management system, there was flexibility for clinical debate. In the context of scientific uncertainty and needing to evaluate the murky waters of neuroscience hypotheses and research, discussion and debate were strengths which could help ensure a diversity of approaches with no single new method of clinical treatment being able to dominate practice. It could, however, contribute to the leadership’s over-caution and conservatism verging on complacency about making changes. Combined with paternalism, a preoccupation with budgets, obedience to higher authorities and to the lunacy legislation, the style of leadership contributed to sluggish responses in the face of changing needs and circumstances.

The Board kept its head down, usually complied with demands from above and only rarely advocated for patients in its asylums. Despite some openness from the leadership about science and psychiatry, it is hard to conclude with a contextualised and respectful analysis of the asylum management system. It was secretive, self-protective, shady, patronising, rejecting of ideas from outside (except from seniority or science), censorious of staff lower in the hierarchy, and neglectful of patients, despite care and treatment of those patients being the stated rationale of the asylums.

## Patients, Outcomes and Austerity

Providing appropriate individualised care and treatment was influenced by powerful stakeholders who held diverse values and objectives and too often cut corners and services. Ongoing frugality in asylum management culture was particularly evident in the context of competing priorities associated with wartime austerity. The wartime asylums were characterised by a decline in standards rather than a cliff-edge change. Many defects pre-war became increasingly hazardous as the war progressed. Food, nutrition, fuel, hygiene, overcrowding, understaffing, staff discontent , and medical attention to patients were some of the aspects which deteriorated. The result was disastrous from the point of view of patient wellbeing.

Clinical notes reveal severe mental and physical illness in asylum patients which caused much suffering and disability. Some people entered asylums with rapidly fatal diseases, some were discharged (whether or not fully recovered), and others stayed as patients until they died months or years later, too often from potentially preventable infectious diseases. Despite recent popular fiction featuring women incarcerated for no other reason than giving birth to an illegitimate child, this was rare.^14^ Neither did asylums seek to admit people purely because they were socially “impossible, inconvenient or inept” to create “Warehouses of the Unwanted”, as Andrew Scull described.^15^ Some troublesome people were dumped by families who had done all they could and had reached a point of despair, coping with an impossible domestic situation with insufficient guidance and support, but this does not mean that the patients were “unwanted”. Others were dumped from within the healthcare system, particularly patients who had serious physical illness complicated by hallucinations, delusions and disturbed behaviours, likely to have been due to delirium .^16^ Transferring these physically ill patients to asylums from other institutions, particularly workhouse infirmaries or general hospitals, was medically illogical. The practice reflected the ongoing attitudes of many non-asylum doctors, to get a “hopeless” patient, especially if perceived as senile or delirious, off their hands as rapidly as possible.^17^

Despite the total number of asylum patients declining, mainly due to high death rates and fewer admissions, overcrowding worsened, associated with more custodial care and fewer discharges, linked to reduced bed availability for civilian patients due to asylums being converted into war hospitals. The reduced admission rates were multifactorial, likely to have been associated with: greater social cohesion in the face of national adversity; reduced alcohol intake; some men being admitted without certification to military mental hospitals; and awareness by the magistrates and doctors who oversaw admissions that there were fewer beds and standards had dropped.^18^

As opposed to aiming for prolonged detention, discharging patients from asylums as soon as possible was vital, to vacate beds to allow admission of new, acutely unwell, patients. Around 40 per cent of patients were discharged within a year of admission in the late Victorian era, but this rate declined when asylums filled up with many long-term, chronically ill people, and custodial care replaced more active, individualised treatment. By 1918, the discharge rate had fallen by one third.^19^ The chronic course of many severe mental disorders, insufficient rehabilitative treatment combined with the Lunacy Act’s cumbersome bureaucratic discharge procedures, and an excessively cautious approach to determining whether a patient might still be dangerous to themselves or to others, all contributed to obstructing discharge. With vague disease classification, and the Board’s annual report for 1918 preoccupied with causes of death rather than of admissions, it is not possible to determine whether there were any significant changes in the types of mental disorder for which patients were admitted (except related to alcohol intake) which might have affected outcomes during the war. From the evidence available, it is likely that overcrowding and understaffing worsened from their pre-war levels and did not allow sufficient therapeutic attention to promote recovery. Other factors which contributed to custodial batch-living, rather than active and rehabilitative treatment, included poor staff morale and questionable methods of placing patients on wards according to their behaviours, for organisational convenience, rather than linked to identified cause, or likely treatment requirements, or expected prognosis.

There was also a lack of after-care. One argument used against providing it was that former patients would not want any assistance which might reveal their asylum admission and pauper lunatic status to their local community, as it might lead to them being ostracised. This opinion, from some of the leadership, was convenient and in line with maintaining the *status quo*, but the Mental After Care Association’s (MACA) papers suggest that the argument was flawed. MACA’s archives may be biased in their own favour, but they nevertheless reveal that patients valued MACA’s support, and that the charity had to turn people away as demand exceeded means. Generalisations by the asylum leadership about patients’ views revealed their own negativity and lack of understanding of insanity. Concerning after-care, even if a patient’s judgement was assumed to be inevitably impaired while they were mentally unwell, by definition, at the time of discharge their judgement would have recovered alongside their sanity, and their views should have been attended to. Negative attitudes towards insanity, not listening to patients, and a persistent desire to minimise short-term expenditure, were hardly ideal qualities for management teams supposedly working in the patients’ best interests.

The way in which the asylum authorities dealt with the rising death rate from potentially preventable infections is also disturbing. Guidance was circulated to asylums for over a decade pre-war, based on scientific and public health evidence about what ought to be tackled to minimise the spread of infections, particularly tuberculosis, but implementation was neglected. At the beginning of the war, the asylum annual death rate from all causes hovered around 10 per cent. In 1915 the Board showed no inclination to investigate when deaths had risen to an unprecedented high of 12 per cent (Table 10.1007/978-3-030-54871-1_7). By the end of the war the death rate was 20 per cent, with relatively little of that due to the influenza pandemic. The general population, despite poverty and hardship, did not suffer the high rates of infectious diseases of patients in the asylums, before or during the war. The huge peak of tuberculosis deaths in the wartime asylums was multifactorial,^20^ but included neglect.

Overall, patients suffered not just because of the disorders which led to their admission but because of the way the institutions were managed before and during the war. No doubt many people did their best, despite ambiguous science for mysterious and frightening mental disorders which often ran a chronic course. However, the leadership’s negative attitudes towards the people they were meant to serve, and, among other things, their rigidity, complacency and penny-pinching, impaired their patients’ health and wellbeing, with death rates from preventable disorders far in excess of those in the community. Fighting the war was a necessity, but it is questionable whether the degree of asylum neglect was necessary, justifiable or compatible with basic principles of medical ethics.^21^

Failure to prevent and treat physical disorders suffered by mentally unwell people was not just a feature of the Edwardian era and the First World War: it happens today. As a century ago, diet, lifestyle and late diagnosis of physical disorders continue to contribute to inequalities in life expectancy for people with serious chronic mental illnesses.^22^ The physical disorders in 2020 are primarily cardiovascular disease and diabetes,^23^ different from those a century ago. It is conceivable, as some of our forebears argued, that people with severe mental illnesses also have a biological susceptibility to life-shortening disorders, but it is unlikely, as the types of disorders over time are so different. It is more likely that the acquisition of the various physical problems were, and are, associated with poverty, deprivation, lifestyle and other external risk factors, whether in the asylum or community. In 2018, a study of physical health problems in people with bipolar disorder and schizophrenia concluded that the mortality gap between them and the general population was widening.^24^ There is recognition that people with these mental disorders can benefit from support to make healthy lifestyle changes,^25^ but during the last decade of austerity, the increasing mortality gap suggests that resources are insufficient or ineffective. It is disturbing that any parallels can be drawn between the potentially preventable physical diseases experienced in First World War asylums and in mental healthcare in 2020.

## Making Change

Public support for mentally disturbed soldiers was heartening. It initially helped the soldiers receive more dignified standards of care than those provided for mentally disturbed civilian patients, and it had the potential to encourage good care for all patients with mental disorders. During the war, public support extended to civilian patients as far as assisting the London County Council to change the designation of its institutions from “asylum” to “hospital”.^26^ This was an important symbolic step towards how the leadership intended the asylums to function, as well as indicating the effect which the public could have on the authorities for making change. Public opinion, however, was not always welcome: in the judgement of those in authority, including the Board, trained personnel with scientific, clinical and legal knowledge already knew what to do and how to do it.

 Shell shock reinforced earlier understanding that mentally disturbed people could recover and that benefits could be derived from early treatment, although the Lunacy Act obstructed that for civilians. Shell shock also encouraged new psychological methods of treatment, but those were only accessible to people who could afford private care because they required staff time, impractical in overcrowded and understaffed asylums. Having shell shocked patients in the asylums highlighted inadequacies of provision for their civilian counterparts, but the soldiers’ special status, clothing and privileges also caused problems. These could detract from plans to improve the lives of civilian patients, such as by the gambling, jealousy and theft associated with soldier patients receiving half-a-crown a week, discouraging the authorities from introducing cash remuneration for working patients. This study points to the importance of pre-war ideals and psycho-social, cultural, administrative, financial, clinical and other factors arising from inside the civilian asylum system during the war, as slowly, but erratically, leading to changes in asylum culture and practice. Post-war, rather than changing asylums for the better, shell shock was swallowed up into it, with many long-term civilian and soldier patients treated similarly, side by side in the asylums.^27^

Reduced asylum bed occupancy after the war, particularly when the war hospitals began to revert to their pre-war use, diminished any sense of urgency to provide more or better facilities or to expand services, such as after-care, to meet civilian patients’ needs. Post-war inflation also detracted from improving standards in the asylums. The cost of treating a patient in an asylum in 1921 was more than double that in 1914, necessitating lifting the Lunacy Act’s cap on charges payable by the Boards of Guardians.^28^ This rise mainly covered costs of higher staff salaries, reduced hours of work, and improved working conditions. Undoubtedly, these measures had the potential to improve care for patients. However, that was not their purpose. They were a response to the National Asylum Workers’ Union (NAWU) campaign since 1918, cotemporaneous with the increased influence of trade unions on workers’ lives. Spending more of the asylum budget on staffing risked reducing the amount spent directly on patients.

The Board was initially ambivalent towards establishing a Ministry of Health, partly as it was concerned about protecting its own role. Nevertheless, it eventually welcomed its transfer, and that of the asylums, from the Home Office to the new Ministry in 1919.^29^ The Board reasonably expected the move to help “dispel prejudices which often arise against Lunacy authorities and administrations, and which often affect injuriously, patients under treatment or even after recovery.”^30^ Whether it did that, or to what degree, is outside the scope of the present study, but the move brought mental and physical illnesses closer together for administrative purposes. Alongside changing “asylum” to “hospital”, it was another important step on the long path towards “parity of esteem”, to fund services for people with mental and physical illnesses in proportion to the morbidity which they cause, a goal still not achieved.^31^ Despite the move to the new Ministry, other branches of healthcare—public health, maternal and child health, medicine and surgery—remained priorities on professional, public and government agendas, as judged by recurring themes in the *Lancet*^32^ and the concerns of the Reconstruction Committee. In 1920, the Minister of Health, Christopher Addison, introduced a bill into parliament covering a diversity of health-related needs, one of which was to permit voluntary admission to public asylums. The bill was rejected by the Lords, much to the disappointment of the Board.^33^

A tricky situation was that the Board only had the authority to recommend change rather than to enforce it. The Board suggested, cajoled, named and shamed, and used any other technique it could to persuade asylums to raise standards. Tactics of persuasion could succeed but were most likely to do so with the most motivated. Too often, the Board’s informal approach, trusting the VCs and medical superintendents to do what was asked in the interests of the patients, did not work. The Board, for example, had repeatedly prompted the VC at Prestwich to replace patients’ earth closets with water closets. This was only implemented when the deficit came under public scrutiny at the Cobb Inquiry in 1922, as a result of Montagu Lomax’s book about his wartime asylum experiences.^34^

Witnesses at the Cobb Inquiry revealed many defects in asylum care, treatment and facilities, but the inquiry report concluded that “the care and treatment of the insane is humane and efficient” and “compares favourably with that in any other country”. The first of these statements is incompatible with much evidence presented at the inquiry by former patients and lower tiers of staff. The second is relative and raises questions about how it was derived, since the inquiry did not evaluate international evidence. Notwithstanding the report’s reassurance, it also stated that there were “certain directions in which improvements and developments could be effected with advantage. It is of course obvious that these would involve increased expenditure”, for which the community had responsibility.^35^ It was good that Cobb acknowledged the importance of the public for making changes, but it was unfortunate that the country was in the midst of a financial crisis and that greater involvement would require major organisational and culture shifts by the public and the leadership, neither of which were on the horizon.

Cobb’s report was not alone in its pattern of negating evidence from patients and lower ranks, reassuring the responsible authorities of the adequacy of their leadership, and then countering its own conclusions by arguing for improvements. In particular, it resembled the responses of the committees of inquiry into the *Sans Everything* allegations of scandalous care of elderly people in National Health Service long-stay geriatric and psychiatric wards four decades later.^36^ Ultimately, the *Sans Everything* inquiries led to many improvements. Similarly, follow-up of the Cobb Report included a Royal Commission which led to the more patient-focussed Mental Treatment Act 1930.^37^

Forty years after the Lunacy Act and 25 after the first parliamentary attempt to reform it, the Mental Treatment Act permitted early and voluntary admission to the mental hospitals in line with long-term psychiatric understanding of its likely health benefits. This achievement and its implications meant that, more directly than psychological and clinical understanding derived from shell shock or from research into psychiatric disorders, Lomax’s book stimulated processes which ultimately liberalised rules on admission and shaped treatment for patients in mental hospitals across the country.

## Final Word

It is easy to imagine a nurse or attendant a century ago expressing sentiments similar to those of an anonymous mental health nurse in the *Guardian* in 2019:

I’m a mental health nurse. There are no good decisions, only least bad ones. I often feel I’m letting my patients down, but I do this job because I believe in the healing power of small acts of kindness…. My day off. I go to the pub and see my friends, who make effort to give me space to talk about work. My answers are scant, because it would drain us all to go into detail, and I just want to enjoy my pint. I work in close proximity to so much suffering that I can never quite find the language to explain it all.^38^

Despite the problems in the asylums we must remember much care and many kindnesses. Kindness from staff members was assumed so it was not noteworthy. It was rarely mentioned specifically in official records, only coming to light in the context of some other pressing matter. Within the asylums we know about Nurse H’s remorse for injuring Edith B, compassion shown to Louise F at Claybury, Eliza Maidman’s loyalty to her asylum, and acting medical superintendents Guy Barham and Alfred Daniel who spoke up to provide better treatment for their patients. Colney Hatch sought to provide for the religious, language and cultural needs of the East End Jewish community, Belgian refugees, prisoners of war and others. Some patients had a sense of community with meaningful relationships within their asylum home, and maintained strong bonds with their families.

Outside the asylums, the Home Office refused permission to deport Mayer L, MACA pioneered individualised rehabilitation programmes, parliamentarians in both Houses challenged the government about inadequate provision for civilian patients with mental disorders, and Mercier, Stoddart, Weatherly and others advocated forcefully and repeatedly for humane and therapeutic treatment and lunacy law reform. We must be grateful too to the lower ranks of staff and the patients who stood their ground to say what needed to be said, especially at the Cobb Inquiry, and to a handful of patients, such as Mary Riggall, James Scott and Rachel Grant-Smith, who revealed their personal stories of asylum life, good and bad, with a view to encouraging change for the better.

### Notes

1. Rab Houston, “Past and ‘Pastism’ in the History of Psychiatry,” *Lancet Psychiatry* 6 (2019): 206–8.
2. E.g. Charles Mercier, *The Attendant’s Companion: A Manual of the Duties of Attendants in Lunatic Asylums* (London: J and A Churchill, 1898); William Stoddart, *Mental Nursing* (London: Scientific Press, 1916); Lionel Weatherly, *A Plea for the Insane: The Case for Reform in the Care and Treatment of Mental Diseases* (London: Grant Richards Ltd, 1918).
3. *First Annual Report of the Board of Control, for the Year 1914* (*BoC AR 1914*) (London: HMSO, 1916), Part 2, Brecon and Radnor Asylum 6 May 1914, 199.
4. Diane Carpenter, “‘Above All a Patient Should Never Be Terrified’: An Examination of Mental Health Care and Treatment in Hampshire 1845–1914” (PhD thesis, University of Portsmouth, 2010), https://researchportal.port.ac.uk/portal/files/5877161/Diane_Carpenter_PhD_Thesis_2010.pdf, 156, 158; *BoC AR 1914*, Part 2, The Chestnuts, Walthamstow, 28 October 1914, 228.
5. SANE, *Schizophrenia and Employment: Putting the Lived*-*Experience of Schizophrenia at the Heart of the Employment Agenda*, 2013, http://www.sane.org.uk/uploads/schizophrenia_employment_web.pdf; Office for National Statistics, *Statistical Bulletin: Labour Market Statistics*, April 2013, http://www.ons.gov.uk/ons/dcp171778_305051.pdf.
6. Schizophrenia Commission, *The Abandoned Illness*, 2012: 6, https://www.rethink.org/media/514093/TSC_main_report_14_nov.pdf.
7. Claybury LCC/MIN/00949 Meeting, 20 June 1918, 86–88 LMA.
8. Mental Health Foundation, *The COVID*-*19 Pandemic, Financial Inequality and Mental Health*, 2020, https://www.mentalhealth.org.uk/sites/default/files/MHF-covid-19-inequality-mental-health-briefing.pdf.
9. House of Commons Committee of Public Accounts, *Improving Access to Mental Health Services*, Sixteenth Report of Session 2016–2017, HC 80, https://publications.parliament.uk/pa/cm201617/cmselect/cmpubacc/80/80.pdf.
10. Colney Hatch LCC/MIN/01001 Meeting, 23 May 1913, 95–96 LMA; Napsbury H50/A/01/024, Meeting, 5 November 1915, 249 LMA.
11. Claire Hilton, *Improving Psychiatric Care for Older People: Barbara Robb’s Campaign 1965*–*1975* (London: Palgrave Macmillan, 2017).
12. John Martin with Debbie Evans, *Hospitals in Trouble* (Oxford: Blackwell, 1984); Louise Hide and Joanna Bourke, “Cultures of Harm in Institutions of Care: Introduction,” *Social History of Medicine* 31 (2018): 679–87.
13. Adolph Meyer, “The Aims of a Psychiatric Clinic,” 1–11, in *XVIIth International Congress of Medicine, London 1913. Section XII Psychiatry. Part 1* (London: Henry Fowde, Hodder and Stoughton, 1913), 1–2.
14. E.g. Sebastian Barry, *The Secret Scripture* (London: Faber and Faber, 2008); Maggie O’Farrell, *The Vanishing Act of Esme Lennox* (London: Headline Review, 2006).
15. Andrew Scull, *The Most Solitary of Afflictions: Madness and Society in Britain, 1700*–*1900* (New Haven: Yale University Press, 1993), 370.
16. Claybury LCC/MIN/00948 Meeting, 3 January 1918, 286 LMA.
17. Tom Arie, “Dementia in the Elderly: Diagnosis and Assessment,” *BMJ* 1 December 1973, 540–43, 541.
18. *BoC AR 1914*, Part 1, 10; *Sixth Annual Report of the Board of Control, for the Year 1919* (*BoC AR 1919*) (London: HMSO, 1920), 10; *Seventh Annual Report of the Board of Control, for the Year 1920* (*BoC AR 1920)* (London: HMSO, 1921), Appendix A, 87.
19. *BoC AR 1919*, Appendix A, 22–23.
20. John Murray, “Tuberculosis and World War I,” *American Journal of Respiratory and Critical Care Medicine* 192 (2015): 411–14.
21. John Keay, “Presidential Address on the War and the Burden of Insanity,” *Journal of Mental Science* 64 (1918): 325–44.
22. King’s Fund, *Mental Health: Our Position*, 2019, https://www.kingsfund.org.uk/projects/positions/mental-health.
23. NHS England, *Improving the Physical Health of People with Serious Mental Illness: A Practical Toolkit*, 2016, https://www.england.nhs.uk/mentalhealth/wp-content/uploads/sites/29/2016/05/serious-mental-hlth-toolkit-may16.pdf.
24. Joseph Hayes, Louise Marston, Kate Walters, Michael King and David Osborn, “Mortality Gap for People with Bipolar Disorder and Schizophrenia: UK-Based Cohort Study 2000–2014,” *British Journal of Psychiatry* 211 (2017): 175–81.
25. Sarah Barber and Graham Thornicroft, “Reducing the Mortality Gap in People with Severe Mental Disorders: The Role of Lifestyle Psychosocial Interventions,” *Frontiers in Psychiatry*, 28 September 2018, 10.3389/fpsyt.2018.00463.
26. LCC LCC/MIN/00583 Meeting, 18 December 1917, 234–36 LMA.
27. Peter Barham, *Forgotten Lunatics of the Great War* (New Haven and London: Yale University Press, 2004), 297.
28. *Eighth Annual Report of the Board of Control, for the Year 1921* (London: HMSO, 1922), 27–28.
29. Ministry of Health Act 1919 section 3.
30. *BoC AR 1920*, 1.
31. Claire Hilton, “Parity of Esteem for Mental and Physical Health Care in the United Kingdom: A Hundred Years War?” *Journal of the Royal Society of Medicine* 109 (2016): 133–37.
32. E.g. Anon. “Cerebellar Syndrome Following Heat Stroke,” *Lancet* 26 October 1918, 561–62; Anon. “Lethargic Encephalitis and Poliomyelitis,” *Lancet* 28 December 1918, 888; Anon. “Standardisation of Pathological Methods,” *Lancet* 26 October 1918, 563; Anon. “Botulism Due to Canned Vegetables,” *Lancet* 16 November 1918, 677; Walter Spencer, “Caesarean Section Three Times on the Same Patient,” *Lancet* 7 December 1918, 778–79.
33. Ministry of Health (Miscellaneous Provisions) Bill. *Hansard* HL Deb 14 December 1920 vol 39 cc119-49; *BoC AR 1920*, 2.
34. Committee on the Administration of Public Mental Hospitals (Chairman: Sir Cyril Cobb) (Cobb Inquiry); Montagu Lomax, *The Experiences of an Asylum Doctor* (London: Allen and Unwin, 1921).
35. Ministry of Health, *Report of the Committee on Administration of Public Mental Hospitals* Cmd. 1730 (London: HMSO, 1922), 80.
36. Mental Health Service. *Hansard* HC Deb 19 March 1965, vol. 708 cc.1645–1719; Ministry of Health, *Findings and Recommendations Following Enquiries into Allegations Concerning the Care of Elderly Patients in Certain Hospitals* Cmnd. 3687 (London: HMSO, 1968); Claire Hilton, *Improving Psychiatric Care for Older People: Barbara Robb’s campaign 1965*–*1975* (London: Palgrave Macmillan, 2017).
37. Hilton, *Improving Psychiatric Care*; *Report of the Royal Commission on Lunacy and Mental Disorder* Cmd. 2700 (London: HMSO, 1926).
38. Anon. “I’m a Mental Health Nurse: There Are No Good Decisions, Only Least Bad Ones,” 11 November 2019, https://www.theguardian.com/society/2019/nov/11/mental-health-nurse-no-good-decisions-patients.

